# Supplementary material for: Assessment of Waterbird Habitat Importance and Identification of Conservation Gaps in Anhui Province
Source: Animals (Basel). 2024 Mar 25;14(7):1004. doi: 10.3390/ani14071004 (PMC11011108; doi:10.3390/ani14071004)
Supplement: Supplementary file 1 [file animals-14-01004-s001.zip › Supplementary material-Final Proofreading Before Publication/Supplementary material-Final Proofreading Before Publication.doc]

**Table S1: List of important habitats for waterbirds in Anhui Province**

| **Wetland** | **Importance scores** | | | | **Total importance score** | **Level** |
| --- | --- | --- | --- | --- | --- | --- |
|  | **Spring migration period** | **Breeding period** | **Autumn migration period** | **Wintering period** |  |  |
| Shengjin Lake | 8.6119 | 1.277 | 7.6657 | 7.2198 | 24.7745 | Level I |
| Huangpi Lake | 3.4544 | 8.2398 | 3.578 | 3.5774 | 18.8495 | Level I |
| Wuchang Lake | 3.9525 | 3.3592 | 6.3113 | 2.6653 | 16.2883 | Level I |
| Caizi lakes | 5.4384 | 0.3021 | 3.2471 | 6.6742 | 15.6618 | Level I |
| Huanghu Lake | 3.8579 | 2.442 | 2.1026 | 4.9567 | 13.3591 | Level II |
| Daguan Lake | 4.7871 | 3.5628 | 1.2416 | 1.553 | 11.1444 | Level II |
| Longguan Lake | 2.5001 | 1.0468 | 2.8962 | 3.0777 | 9.5208 | Level II |
| Chenyao Lake | 3.3422 | 0.0331 | 2.6237 | 2.7761 | 8.7752 | Level II |
| Po Lake | 1.4949 | 0.2872 | 1.8403 | 3.7495 | 7.372 | Level II |
| Pihe River | 0.1212 | 5.3441 | 1.1347 | 0.3106 | 6.9106 | Level II |
| Chaohu Lake | 0.8081 | 0.2765 | 3.111 | 1.4005 | 5.5961 | Level III |
| Chengxi Lake | 0.3643 | 0.3633 | 1.1552 | 2.7228 | 4.6056 | Level III |
| Jiaogang Lake | 2.6432 | 0.019 | 1.0771 | 0.4895 | 4.2288 | Level III |
| Shijiu Lake | 0.6115 | 0.0662 | 1.8464 | 1.6394 | 4.1635 | Level III |
| Bali River | 0.7621 | 0.1401 | 0.1667 | 2.4024 | 3.4712 | Level III |
| Wanhe River | 0.2043 | 0.6911 | 1.3046 | 1.2655 | 3.4656 | Level III |
| Tuohu Lake | 0.5461 | 0.0431 | 1.8586 | 0.9292 | 3.377 | Level III |
| Nvshan Lake | 1.1106 | 0.2379 | 0.6747 | 1.3003 | 3.3234 | Level III |
| Shibasuo | 1.0193 | 0.0478 | 0.1222 | 1.6561 | 2.8454 | Level III |
| Wangjiaba | 0.9841 | 0.6009 | 0.0669 | 1.1236 | 2.7756 | Level III |
| Chengdong Lake | 0.8192 | 0.3417 | 0.6836 | 0.8973 | 2.7418 | Level III |
| Lianjiangba Reservoir | 0.8158 | 0.0237 | 1.067 | 0.8205 | 2.7271 | Level III |
| Qili Lake | 0.319 | 0.169 | 0.6197 | 1.5169 | 2.6245 | Level III |
| Dunziwang Reservoir | 1.6824 | 0.0371 | 0.0403 | 0.5853 | 2.345 | Level III |
| Baidang Lake | 0.905 | 0.0344 | 0.825 | 0.5296 | 2.294 | Level III |
| Sifang Lake | 0.5688 | 0.6419 | 0.5404 | 0.3878 | 2.1388 | Level III |
| Zhusi Lake | 0.9121 | 0.1443 | 0.3664 | 0.6992 | 2.122 | Level III |
| Xiangjian Lake | 0.5284 | 0.043 | 0.4681 | 1.0537 | 2.0932 | Level III |
| Dongpi River | 0.4927 | 0.0934 | 0.5359 | 0.6357 | 1.7576 | Level IV |
| Fushan Reservoir | 0.7737 | 0.0308 | 0.8176 | 0.0775 | 1.6996 | Level IV |
| Fengsha Lake | 0.6811 | 0.0332 | 0.4197 | 0.5637 | 1.6977 | Level IV |
| Gangyao Lake | 0.919 | 0.0913 | 0.1055 | 0.4015 | 1.5173 | Level IV |
| Wanfohu Reservoir | 0.1037 | 0.0258 | 0.1671 | 1.0901 | 1.3867 | Level IV |
| Caicheng Lake | 0.1479 | 0.2767 | 0.1829 | 0.7255 | 1.3331 | Level IV |
| Nanyi Lake | 0.057 | 0.4229 | 0.0842 | 0.7504 | 1.3144 | Level IV |
| Shuiqiao Lake | 0.0413 | 0.0438 | 0.0574 | 1.1381 | 1.2806 | Level IV |
| Minghu Park | 1.0357 | 0.0298 | 0.0207 | 0.123 | 1.2092 | Level IV |
| Shiwan Reservoir | 0.8796 | 0.0624 | 0.0421 | 0.17 | 1.154 | Level IV |
| Qiupuheyuan | 0.1111 | 0.0219 | 0.9848 | 0.0101 | 1.128 | Level IV |
| Qianshui River | 0.05 | 0.0835 | 0.3324 | 0.5701 | 1.036 | Level IV |
| Huayuan Lake | 0.0333 | 0.2717 | 0.4286 | 0.2779 | 1.0115 | Level IV |
| Zhegao River | 0.0532 | 0.6984 | 0.1454 | 0.0754 | 0.9724 | Level IV |
| Chengbei Reservoir | 0.7489 | 0.0328 | 0.0372 | 0.135 | 0.954 | Level IV |
| Anle Reservoir | 0.7937 | 0.0619 | 0.0601 | 0.0344 | 0.9501 | Level IV |
| Huajia Lake | 0.1043 | 0.0714 | 0.5052 | 0.2275 | 0.9084 | Level IV |
| Nandian Reservoir | 0.5031 | 0.0349 | 0.1421 | 0.208 | 0.8881 | Level IV |
| Langan Lake | 0.373 | 0.0291 | 0.4205 | 0.0435 | 0.866 | Level IV |
| Qianhewa | 0.0954 | 0.0459 | 0.6423 | 0.0744 | 0.858 | Level IV |
| Tianhe Lake | 0.0548 | 0.2634 | 0.0589 | 0.4642 | 0.8414 | Level IV |
| Fengshouwei | 0.0796 | 0.0639 | 0.0394 | 0.6513 | 0.8342 | Level IV |
| Snhe River | 0.1757 | 0.2529 | 0.314 | 0.0891 | 0.8316 | Level IV |
| Bandao Wetland Park | 0.0845 | 0.3577 | 0.2417 | 0.1444 | 0.8283 | Level IV |
| Huailin Wetland Park | 0.0509 | 0.254 | 0.1571 | 0.3302 | 0.7921 | Level IV |
| Tiansheng Lake | 0.1846 | 0.0445 | 0.1543 | 0.3424 | 0.7258 | Level IV |
| Zhongxing Reservoir | 0.2499 | 0.0416 | 0.146 | 0.2805 | 0.718 | Level IV |
| Datong Reservoir | 0.4542 | 0.0559 | 0.0508 | 0.1439 | 0.7047 | Level IV |
| Caiqiao Reservoir | 0.5435 | 0.0351 | 0.056 | 0.0591 | 0.6936 | Level IV |
| Yihu Lake | 0.0522 | 0.4726 | 0.0419 | 0.1194 | 0.6861 | Level IV |
| Chengxi Reservoir | 0.0602 | 0.0344 | 0.0481 | 0.5179 | 0.6606 | Level IV |
| Wabu Lake | 0.0587 | 0.0678 | 0.3912 | 0.1331 | 0.6508 | Level IV |
| Sanwan Reservoir | 0.1388 | 0.4416 | 0.0269 | 0.038 | 0.6452 | Level IV |
| Chishan Lake | 0.0478 | 0.4659 | 0.0279 | 0.1023 | 0.6439 | Level IV |
| Huating Lake | 0.1516 | 0.059 | 0.2228 | 0.2103 | 0.6437 | Level IV |
| Taiping Lake | 0.2689 | 0.0374 | 0.1339 | 0.1794 | 0.6197 | Level IV |
| Randeng Reservoir | 0.4357 | 0.0495 | 0.0592 | 0.0709 | 0.6154 | Level IV |
| Qiqiao Reservoir | 0.0351 | 0.0538 | 0.1352 | 0.3888 | 0.6129 | Level IV |
| Anfengtang | 0.1469 | 0.0254 | 0.3683 | 0.0482 | 0.5889 | Level IV |
| Sangjian Reservoir | 0.5062 | 0.0255 | 0.0266 | 0.0262 | 0.5845 | Level IV |
| Qingyi River | 0.0827 | 0.3851 | 0.0485 | 0.0392 | 0.5555 | Level IV |
| Changjiang River | 0.0384 | 0.4668 | 0.0225 | 0.0229 | 0.5506 | Level IV |
| Pogang Lake | 0.0698 | 0.101 | 0.0627 | 0.3167 | 0.5501 | Level IV |
| Hengjiang River | 0.059 | 0.0511 | 0.0557 | 0.3712 | 0.5371 | Level IV |
| Pingtian Lake | 0.4599 | 0.0101 | 0.0311 | 0.0331 | 0.5342 | Level IV |

**Table S2: List of waterbirds in Anhui Province**

| **Order** | **Family** | **Species** | **National protection level** | **IUCN** |
| --- | --- | --- | --- | --- |
| ANSERIFORMES | Anatidae | *Cygnus cygnus* | II | LC |
| ANSERIFORMES | Anatidae | *Cygnus columbianus* | II | LC |
| ANSERIFORMES | Anatidae | *Anser caerulescens* |  | LC |
| ANSERIFORMES | Anatidae | *Anser indicus* |  | LC |
| ANSERIFORMES | Anatidae | *Anser anser* |  | LC |
| ANSERIFORMES | Anatidae | *Anser coygnoid* | II | VU |
| ANSERIFORMES | Anatidae | *Anser fabalis* |  | LC |
| ANSERIFORMES | Anatidae | *Anser albifrons* | II | LC |
| ANSERIFORMES | Anatidae | *Anser erythropus* | II | VU |
| ANSERIFORMES | Anatidae | *Bucephala clangula* |  | LC |
| ANSERIFORMES | Anatidae | *Mergellus albellus* | II | LC |
| ANSERIFORMES | Anatidae | *Mergus merganser* |  | LC |
| ANSERIFORMES | Anatidae | *Mergus squamatus* | I | EN |
| ANSERIFORMES | Anatidae | *Tadorna tadorna* |  | LC |
| ANSERIFORMES | Anatidae | *Tadorna ferruginea* |  | LC |
| ANSERIFORMES | Anatidae | *Nettapus coromandelianus* | II | LC |
| ANSERIFORMES | Anatidae | *Aix galericulata* | II | LC |
| ANSERIFORMES | Anatidae | *Netta rufina* |  | LC |
| ANSERIFORMES | Anatidae | *Aythya ferina* |  | VU |
| ANSERIFORMES | Anatidae | *Aythya baeri* | I | CR |
| ANSERIFORMES | Anatidae | *Aythya nyroca* |  | NT |
| ANSERIFORMES | Anatidae | *Aythya fuligula* |  | LC |
| ANSERIFORMES | Anatidae | *Aythya marila* |  | LC |
| ANSERIFORMES | Anatidae | *Spatula querquedula* |  | LC |
| ANSERIFORMES | Anatidae | *Spatula clypeata* |  | LC |
| ANSERIFORMES | Anatidae | *Sibirionetta formosa* | II | LC |
| ANSERIFORMES | Anatidae | *Mareca falcata* |  | NT |
| ANSERIFORMES | Anatidae | *Mareca strepera* |  | LC |
| ANSERIFORMES | Anatidae | *Mareca penelope* |  | LC |
| ANSERIFORMES | Anatidae | *Anas zonorhyncha* |  | LC |
| ANSERIFORMES | Anatidae | *Anas platyrhynchos* |  | LC |
| ANSERIFORMES | Anatidae | *Anas acuta* |  | LC |
| ANSERIFORMES | Anatidae | *Anas crecca* |  | LC |
| PODICIPEDIFORMES | Podicipedidae | *Tachybaptus ruficollis* |  | LC |
| PODICIPEDIFORMES | Podicipedidae | *Podiceps cristatus* |  | LC |
| PODICIPEDIFORMES | Podicipedidae | *Podiceps auritus* | II | VU |
| PODICIPEDIFORMES | Podicipedidae | *Podiceps nigricollis* | II | LC |
| PHOENICOPTERIFORMES | Phoenicopteridae | *Phoenicopterus roseus* |  | LC |
| GRUIFORMES | Rallidae | *Rallus indicus* |  | LC |
| GRUIFORMES | Rallidae | *Porzana fusca* |  | LC |
| GRUIFORMES | Rallidae | *Zapornia akool* |  | LC |
| GRUIFORMES | Rallidae | *Amaurornis phoenicurus* |  | LC |
| GRUIFORMES | Rallidae | *Gallinula chloropus* |  | LC |
| GRUIFORMES | Rallidae | *Fulica atra* |  | LC |
| GRUIFORMES | Gruidae | *Leucogeranus leucogeranus* | I | CR |
| GRUIFORMES | Gruidae | *Antigone vipio* | I | VU |
| GRUIFORMES | Gruidae | *Grus japonensis* | I | VU |
| GRUIFORMES | Gruidae | *Grus grus* | II | LC |
| GRUIFORMES | Gruidae | *Grus monacha* | I | VU |
| CICONIIFORMES | Ciconiidae | *Ciconia nigra* | I | LC |
| CICONIIFORMES | Ciconiidae | *Ciconia boyciana* | I | EN |
| PELECANIFORMES | Threskiornithidae | *Platalea leucorodia* | II | LC |
| PELECANIFORMES | Threskiornithidae | *Platalea minor* | I | EN |
| PELECANIFORMES | Ardeidae | *Botaurus stellaris* |  | LC |
| PELECANIFORMES | Ardeidae | *Ixobrychus sinensis* |  | LC |
| PELECANIFORMES | Ardeidae | *Ixobrychus cinnamomeus* |  | LC |
| PELECANIFORMES | Ardeidae | *Ixobrychus flavicollis* |  | LC |
| PELECANIFORMES | Ardeidae | *Nycticorax nycticorax* |  | LC |
| PELECANIFORMES | Ardeidae | *Butorides striata* |  | LC |
| PELECANIFORMES | Ardeidae | *Ardeola bacchus* |  | LC |
| PELECANIFORMES | Ardeidae | *Bubulcus coromandus* |  | LC |
| PELECANIFORMES | Ardeidae | *Ardea cinerea* |  | LC |
| PELECANIFORMES | Ardeidae | *Ardea purpurea* |  | LC |
| PELECANIFORMES | Ardeidae | *Ardea alba* |  | LC |
| PELECANIFORMES | Ardeidae | *Ardea intermedia* |  | LC |
| PELECANIFORMES | Ardeidae | *Egretta garzetta* |  | LC |
| PELECANIFORMES | Pelecanidae | *Pelecanus crispus* | I | NT |
| SULIFORMES | Phalacrocoracidae | *Phalacrocorax carbo* |  | LC |
| CHARADRIIFORMES | Haematopodidae | *Haematopus ostralegus* |  | NT |
| CHARADRIIFORMES | Recurvirostridae | *Recurvirostra avosetta* |  | LC |
| CHARADRIIFORMES | Recurvirostridae | *Himantopus himantopus* |  | LC |
| CHARADRIIFORMES | Charadriidae | *Vanellus vanellus* |  | NT |
| CHARADRIIFORMES | Charadriidae | *Vanellus cinereus* |  | LC |
| CHARADRIIFORMES | Charadriidae | *Pluvialis fulva* |  | LC |
| CHARADRIIFORMES | Charadriidae | *Charadrius placidus* |  | LC |
| CHARADRIIFORMES | Charadriidae | *Charadrius dubius* |  | LC |
| CHARADRIIFORMES | Charadriidae | *Charadrius alexandrinus* |  | LC |
| CHARADRIIFORMES | Rostratulidae | *Rostratula benghalensis* |  | LC |
| CHARADRIIFORMES | Jacanidae | *Hydrophasianus chirurgus* | II | LC |
| CHARADRIIFORMES | Scolopacidae | *Numenius arquata* | II | NT |
| CHARADRIIFORMES | Scolopacidae | *Limosa limosa* |  | NT |
| CHARADRIIFORMES | Scolopacidae | *Calidris temminckii* |  | LC |
| CHARADRIIFORMES | Scolopacidae | *Calidris alpina* |  | LC |
| CHARADRIIFORMES | Scolopacidae | *Gallinago stenura* |  | LC |
| CHARADRIIFORMES | Scolopacidae | *Gallinago gallinago* |  | LC |
| CHARADRIIFORMES | Scolopacidae | *Actitis hypoleucos* |  | LC |
| CHARADRIIFORMES | Scolopacidae | *Tringa ochropus* |  | LC |
| CHARADRIIFORMES | Scolopacidae | *Tringa erythropus* |  | LC |
| CHARADRIIFORMES | Scolopacidae | *Tringa nebularia* |  | LC |
| CHARADRIIFORMES | Scolopacidae | *Tringa totanus* |  | LC |
| CHARADRIIFORMES | Scolopacidae | *Tringa glareola* |  | LC |
| CHARADRIIFORMES | Scolopacidae | *Tringa stagnatilis* |  | LC |
| CHARADRIIFORMES | Glareolidae | *Glareola maldivarum* |  | LC |
| CHARADRIIFORMES | Laridae | *Chroicocephalus ridibundus* |  | LC |
| CHARADRIIFORMES | Laridae | *Saundersilarus saundersi* | I | VU |
| CHARADRIIFORMES | Laridae | *Ichthyaetus relictus* | I | VU |
| CHARADRIIFORMES | Laridae | *Larus argentatus* |  | LC |
| CHARADRIIFORMES | Laridae | *Sterna hirundo* |  | LC |
| CHARADRIIFORMES | Laridae | *Chlidonias hybrida* |  | LC |
| CHARADRIIFORMES | Laridae | *Chlidonias leucopterus* |  | LC |

|  |  |
| --- | --- |
| (a) | (b) |
|  |  |
| (c) | (d) |

**Figure S1: Endangered waterbirds.** (a) *Leucogeranus leucogeranus*; (b) *Ciconia boyciana*; (c) *Aythya baeri*; (d) *Mergus squamatus*.
